# Fungal echinocandin resistance

**DOI:** 10.1016/j.fgb.2009.09.003

**Published:** 2010-02

**Authors:** Louise A. Walker, Neil A.R. Gow, Carol A. Munro

**Affiliations:** School of Medical Sciences, University of Aberdeen, Aberdeen, AB25 2ZD, UK

**Keywords:** *Candida albicans*, *Aspergillus fumigatus*, Antifungals, Fungal cell wall, Glucan, Chitin

## Abstract

The echinocandins are the newest class of antifungal agents in the clinical armory. These secondary metabolites are non-competitive inhibitors of the synthesis of β-(1,3)-glucan, a major structural component of the fungal cell wall. Recent work has shown that spontaneous mutations can arise in two hot spot regions of Fks1 the target protein of echinocandins that reduce the enzyme’s sensitivity to the drug. However, other strains have been isolated in which the sequence of *FKS1* is unaltered yet the fungus has decreased sensitivity to echinocandins. In addition it has been shown that echinocandin-treatment can induce cell wall salvage mechanisms that result in the compensatory upregulation of chitin synthesis in the cell wall. This salvage mechanism strengthens cell walls damaged by exposure to echinocandins. Therefore, fungal resistance to echinocandins can arise due to the selection of either stable mutational or reversible physiological alterations that decrease susceptibility to these antifungal agents.

## 1 Introduction

The fungal cell wall perhaps represents the ideal target for the therapeutic treatment of fungal pathogens of humans. The vast majority of the mass of the cell wall of all fungi is comprised of carbohydrate polymers that are completely absent from the human body. In most fungi, two covalently cross-linked polysaccharides, β-(1,3)-glucan and chitin, form a primary cell wall skeleton that is responsible for structural integrity and shape of the cell (Latge, 2007). This skeletal layer in the inner wall is often surrounded by further polysaccharides composed of mannose, galactose and other sugars that may be covalently attached to cell wall proteins. The complete absence of any one of these glycan polymers is usually lethal to fungal pathogens. Consequently, the enzymes of fungal cell wall polysaccharide biosynthesis are highly specific and potentially cidal targets for antifungal secondary metabolites and antifungal drugs.

Despite the promise of the cell wall as an antifungal target, few classes of cell wall inhibitors have been successfully developed for clinical use. The notable exception is the echinocandins that were discovered in the 1970s by random screening of secondary metabolites. These are cyclic hexapeptides modified with lipid side chains that are essential for their antifungal activity. Caspofungin was the first echinocandin to be approved for clinical use by the FDA followed by anidulafungin and micafungin. The drugs are administered by IV injection and have a relatively broad spectrum of activity with cidal action against most *Candida* species, and either fungicidal or fungistatic action against *Aspergillus fumigatus* (Denning, 2003). They are less effective against *Cryptococcus neoformans*, *Fusarium* and *Scedosporium* although they are able to inhibit growth of *Pneumocystis* (Perlin, 2007). The echinocandins bind to Fks1, a sub-unit of β-(1,3)-glucan synthase, but their binding is non-competitive (Douglas et al., 1997) and formal proof that Fks1 is the catalytic β-(1,3)-glucan synthase is still lacking.

**Fig. 1:**
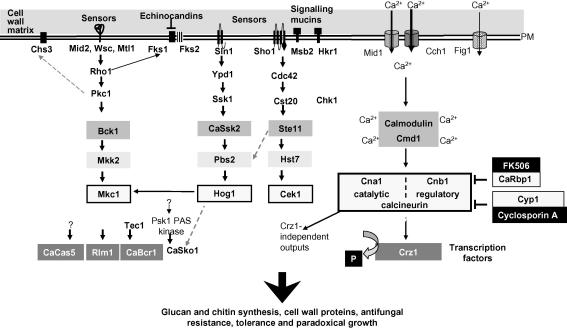
Signalling pathways that regulate cell wall remodelling of *Candida albicans*. The HOG1, CEK1 and PKC MAP kinase cascades and the Ca^2+^–calcineurin signalling pathway control a number of cellular processes including cell wall synthesis and maintenance. Upstream of the MAP kinase cascades are membrane sensors (Wsc family, Mid2, Mtl1, Sho1 and Sln1) that detect alterations in the wall and convey the signal to the internal components of the pathway. The PKC pathway plays a critical role in the response to echinocandins and the first component that is activated is Rho1, which also acts as a regulatory sub-unit of β-(1,3)-glucan synthase. Rho1 activates Protein kinase C, which phosphorylates and activates the MAP kinase kinase kinase Bck1, which in turn activates the MAP kinase kinase Mkk2, which then phosphorylates Mkc1. A number of transcription factors contribute to the response to echinocandins including Cas5 and Sko1. The Rlm1 and Bcr1 transcription factors also control the expression of a number of cell wall related genes. In *S. cerevisiae* Pkc1 is involved in targeting Chs3 to the plasma membrane in response to heat shock. Significant re-wiring of signalling pathways is evident in *C. albicans*, compared to the *S. cerevisiae* paradigm, for example, the role of the CaSko1 transcription factor in response to caspofungin is independent of Hog1 MAP kinase but involves the Psk1 PAK kinase. The calcineurin pathway is activated by calcium that may enter cells through membrane-localised channels Cch1 and Mid1 or a third minor channel Fig. 1, alternatively calcium may be released from intracellular stores. Ca^2+^ binds to and activates calmodulin (Cmd1) that in turn activates the phosphatase calcineurin, which is made up of two sub-units Cna1 and Cnb1. Calcineurin dephosphorylates the transcription factor Crz1, which moves into the nucleus and induces expression of genes through binding to CDREs (calcium dependent response elements) within their promoter sequences. FK506 binding to Fpr1 or cylosporin A binding to cycophilin Cpr1 results in calcineurin inhibition. Adapted from (Levin, 2005; Steinbach et al., 2007b).

In fungi, the integrity of the β-(1,3)-glucan–chitin cell wall scaffold is monitored and regulated constantly to ensure cell viability. This is not a trivial challenge since surface expansion during growth and cellular morphogenesis requires a delicate balance to be maintained between the rigidity and the flexibility of the cell wall. This balance between plasticity and rigidification must also be achievable in the presence of lytic enzymes or antibiotics, such as the echinocandins, that may compromise the integrity of the cell wall. Disruption of genes in cell wall biosynthetic pathways of *Saccharomyces cerevisiae* and *Candida albicans* often results in alteration and redistribution of chitin and β-(1,3)-glucan in the cell wall, synthesis of new wall proteins and changes in the cross-linking to alternative wall polysaccharides (see below). Defects in cell wall integrity are sensed by transmembrane proteins such as Mid2 and the Wsc family leading to activation of the Rho1 GTPase and its downstream targets including protein kinase C and the β-(1,3)-glucan synthase sub-units *Sc*Fks1 and *Sc*Fks2 (Levin, 2005). In *S. cerevisiae*, *C. albicans* and presumably other fungi this “cell wall salvage” or “cell wall compensatory” mechanism is activated in response to wall-perturbing agents such as Calcofluor White (CFW), Congo Red, caffeine and β-glucanases. The response is mediated primarily through the protein kinase C (PKC) cell integrity mitogen-activated protein (MAP) kinase cascade and its downstream target the transcription factor *Sc*Rlm1 (Bermejo et al., 2008; Boorsma et al., 2004; Garcia et al., 2004, 2009; Kuranda et al., 2006; Lagorce et al., 2003). A second MAP kinase cascade, the high osmolarity glycerol response (HOG) pathway, has also been suggested to play a role in regulating cell wall architecture (Bermejo et al., 2008; Garcia-Rodriguez et al., 2000, 2005) (Fig. 1). Finally the Ca^2+^–calcineurin pathway has also been implicated in the activation of cell wall biosynthetic processes in response to damage of the cell wall (Fig. 1) (Lagorce et al., 2003; Walker et al., 2008).

## 2 *FKS1* hot spot mutations confer echinocandin resistance

Global surveillance studies have reported that there is no evidence of any trends towards echinocandin-resistance emerging within clinical isolates of *Candida* species (Pfaller et al., 2006a,b). However, there are a growing number of reports of breakthrough infections in patients receiving echinocandin therapy (Table 1). The mechanism behind the resistance of the clinical isolates responsible for many of these breakthrough infections was first elucidated by *in vitro* studies (Park et al., 2005). Point mutations within the *FKS1* target gene were identified in *C. albicans* strains that were less susceptible to caspofungin after exposure to the drug. Point mutations were then identified in resistant clinical isolates and were clustered around two hot spot regions of the *FKS1* gene (Park et al., 2005; Perlin, 2007). The mutation hot spots map onto amino acids at positions 641–649 (hot spot 1) and 1345–1365 (hot spot 2) of *C. albicans* Fks1. Point mutations within these regions are common to a number of different resistant *C. albicans* clinical isolates with substitution of the serine at position 645 to phenylalanine, proline or tyrosine being the most frequently observed (Balashov et al., 2006; Park et al., 2005; Perlin, 2007). In *C. albicans* these mutations are dominant and confer resistance as heterozygous or homozygous alleles and bestow resistance to all three echinocandins. Enzyme kinetics studies have shown that the sensitivity of the mutated glucan synthase to caspofungin is reduced with K*_i_* increasing from 50-fold to several thousand fold depending upon the mutation (Garcia-Effron et al., 2009b; Park et al., 2005). Garcia-Effron et al., 2009b evaluated the MICs of isolates harboring mutated Fks1 enzymes and found a link between elevated K*_i_* and increased MIC, although this did not necessarily represent a linear relationship.

In *S. cerevisiae* there is an alternative glucan synthase catalytic sub-unit *Sc*Fks2/Gsc2 that is expressed in response to mating pheromone, sporulation and starvation conditions (Mazur et al., 1995). *ScFKS2* is induced by calcium in a calcineurin dependent manner (Mazur et al., 1995). A third related protein *Sc*Fks3 has a role in spore wall assembly (Ishihara et al., 2007). Orthologous proteins are present in other fungal genomes so most fungi contain more than one glucan synthase catalytic sub-unit. Fks1 and its orthologs seem to be the key players during vegetative growth and are the main targets of the echinocandins (discussed below). However, a hot spot mutation has been observed in *S. cerevisiae* Fks2, as well as *Candida guilliermondii* and *Candida glabrata* that resulted in an elevated echinocandin MIC (Garcia-Effron et al., 2009a; Katiyar et al., 2006; Park et al., 2005). Indeed in *C. glabrata FKS2* appears to be more highly expressed than *FKS1* (Garcia-Effron et al., 2009a). Therefore, in some fungal species *FKS2* may contribute more to total glucan synthase activity than *FKS1*.

## 3 Echinocandin resistance of other *Candida* species

Although *C. albicans* is the most common cause of invasive infections other *Candida* species are common agents of disease. In a global surveillance of *Candida* species isolated from blood and other sterile sites from 2001 to 2006 (*n* = 5, 346), 54% were *C. albicans* followed by *Candida parapsilosis* (14%), *C. glabrata* (14%), *Candida tropicalis* (12%), *Candida krusei* (3%) and *C. guilliermondii* (1%) (Pfaller et al., 2008a). The echinocandins are fungicidal against the majority of pathogenic *Candida* species including isolates, which are resistant to azole antifungals.

**Fig. 2:**
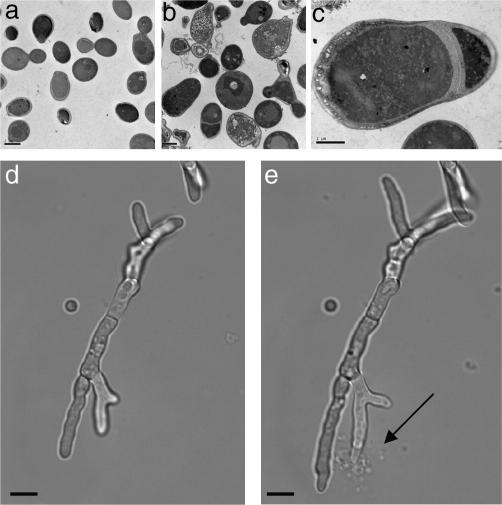
Treatment of fungal cells with caspofungin. Transmission electron micrographs of *Candida albicans* yeast cells grown in YPD medium at 30 °C for 6 h in the absence (a) or presence of 0.032 μg/ml caspofungin (b) and (c). Scale bars represent 2 μm in (a) and (b) and 1 μm in (c). Light microscopy showing a *Aspergillus fumigatus* hypha after 13 h (d) and 14 h (e) treatment with 2 μg/ml caspofungin, a lysed tip is marked by the arrow. In (d) and (e) scale bar is equal to 10 μm.

The minimum inhibitory concentration (MIC) of the echinocandins to *Candida* spp. is determined using a standardised method which has been developed by the Clinical and Laboratory Standards Institute (CLSI) Antifungal Subcommittee. The MIC method uses RPMI-1640 as the test medium containing serial dilutions of drug, which is subsequently inoculated with fungi and incubated for 24 h at 35 °C. The MIC can then be determined by eye or by measuring optical density. The MIC is defined as the point at which there is a ⩾50% inhibition of growth relative to the control. The morphology of *C. albicans* yeast cells grown in the presence of 0.032 μg/ml caspofungin, representing the typical MIC, is shown in Fig. 2, a mixture of live and dead cells are present (Fig. 2b) with some caspofungin-treated cells displaying widened mother-bud necks and septa (Fig. 2c). Recently the CLSI Antifungal Subcommittee determined the MIC break point of susceptibility to the three echinocandins as ⩽2 μg/ml for *Candida* spp*.* (Pfaller et al., 2008b). However, this breakpoint may miss isolates known to have caused breakthrough infections but which have anidulafungin and micafungin MICs between 1 and 2 μg/ml (Garcia-Effron et al., 2009a).

Different *Candida* species have different susceptibilities to caspofungin. The susceptibility of *Candida* species to caspofungin ranges from *C. albicans* as the most sensitive, then *C. glabrata*, *C. tropicalis* and *C. krusei* with *C. parapsilosis* and *C. guilliermondii* being the least susceptible (Barchiesi et al., 2006; Canton et al., 2006; Pfaller et al., 2006b; Walker, Gow, Munro, unpublished). *C. parapsilosis* and *C. guilliermondii* are thought to be intrinsically more resistant to caspofungin due to naturally occurring point mutations in Fks1 (Barchiesi et al., 2006; Canton et al., 2006; Garcia-Effron et al., 2008a). All members of the *C. parapsilosis* family; *C. parapsilosis*, *Candida orthopsilosis* and *Candida metapsilosis* contain a point mutation at amino acid position 660 resulting in a proline to alanine substitution, which is thought to make them intrinsically less susceptible to caspofungin (Garcia-Effron et al., 2008a). Likewise, *C. guilliermondii* isolates contain three amino acid polymorphisms in the first hot spot region, although only the methionine to leucine substitution at position 642 is thought to be important for reduced susceptibility to the echinocandins (Perlin, 2007). Other intrinsically resistant fungal species such as *Neurospora crassa*, *Fusarium solani*, *Fusarium graminearum*, *Fusarium verticilliodes* and *Magnaporthe grisea* are known to contain a point mutation at residue 641, in Fks1, which changes phenylalanine to tyrosine (Ha et al., 2006; Katiyar et al., 2006). This suggests that point mutations in Fks1 may lead to resistance to the echinocandins in a wide range of fungal species. Likewise, *C. guilliermondii* was also found to contain the same V641P substitution in Fks2 (Katiyar et al., 2006). This may explain the intrinsic resistance of *C. guilliermondii* to the echinocandins.

## 4 Echinocandin resistance of *Aspergillus* species

The echinocandins have a largely fungistatic effect against moulds and filamentous fungi such as species of *Aspergillus*. They are cidal to the polarised, growing cell at the hyphal tip and can lead to lysis of the hyphal tip, where the nascent cell wall is less rigid due to the absence of cross-linking between glucan and chitin. As treatment with the echinocandins fails to completely inhibit growth in *Aspergillus* species, it is difficult to determine a clear point of inhibition and consequently an accurate MIC is hard to define. As a result, an alternative method known as the minimum effective concentration (MEC) has been introduced to determine the activity of echinocandins against filamentous fungi. The MEC is defined as the lowest drug concentration at which short, stubby, highly branched hyphae are observed (Arikan et al., 2001; Espinel-Ingroff, 2003; Imhof et al., 2003; Kurtz et al., 1994; Odds et al., 2004). Treatment of *A. fumigatus* with caspofungin leads to lysis of hyphal tips (Fig. 2d and e). This lysis is a result of inhibition of *Af*Fks1, which is involved in synthesising new cell wall glucan at the tips of hyphae (Beauvais et al., 2001; Bowman et al., 2002). Although treatment with caspofungin results in lysis of hyphal tips, viability staining has shown that older sub-apical compartments of *A. fumigatus* hyphae are still viable after exposure to caspofungin (Bowman et al., 2002).

As with the *Candida* species, different species of *Aspergillus* have varying susceptibilities to echinocandins (Antachopoulos et al., 2008; Imhof et al., 2003). Anidulafungin displays the greatest inhibition of growth across the *Aspergillus* spp. compared to caspofungin and micafungin. However, this differential inhibition is negated when susceptibilities are tested in the presence of human serum, which is particularly relevant when testing the efficacy of IV drugs (Paderu et al., 2007). Generally *A. fumigatus*, *Aspergillus terreus* and *Aspergillus flavus* have comparable susceptibilities to all three echinocandins (Antachopoulos et al., 2008). In contrast *Aspergillus niger* has been shown to be considerably more susceptible to caspofungin (MEC of 0.1–0.5 μg/ml) than *A. fumigatus* (MEC of 0.2–6 μg/ml), which is thought to be due to differences in cell wall composition (Imhof et al., 2003). Potential mechanisms of resistance to caspofungin in *A. fumigatus* were highlighted by two classes of laboratory generated mutants with reduced susceptibility to caspofungin (Gardiner et al., 2005). Insertion of a point mutation within the *AfFKS1* gene to generate a S678Y amino acid substitution resulted in an MEC of 4 μg/ml (Gardiner et al., 2005) and introducing a S678P mutation raised the MEC of caspofungin, anidulafungin and micafungin to >16 μg/ml (Rocha et al., 2007), compared to 0.25 μg/ml with the susceptible wild-type strain. Spontaneous caspofungin resistant mutants that were generated by cell wall digestion also displayed reduced sensitivity to the drug independent of alterations in the *AfFKS1* sequence (Gardiner et al., 2005). Expression profiling of these mutants after treatment with caspofungin showed upregulation of genes involved in cell wall biosynthesis/remodelling, structural cell components and transport (Gardiner et al., 2005). Alternatively, a clinical isolate of *A. fumigatus* from a patient who failed caspofungin therapy was resistant due to over-expression of the *AfFKS1* gene (Arendrup et al., 2008). Furthermore, Wetzstein et al., 2007 have reported the emergence of anidulafungin-resistant moulds in two patients.

## 5 Breakthrough infections

Global and nation-wide surveillance projects that have monitored the echinocandin susceptibility of *Candida* spp. isolates, collected since the introduction of this class of drugs into the clinic in 2001, have not observed any emerging trends of resistance (Cuenca-Estrella et al., 2006; Dannaoui et al., 2008; Pfaller et al., 2006a, 2008a). However, since 2005 there have been several case reports published that document failure of caspofungin therapy (Table 1). In general, resistance to one echinocandin confers resistance to all others with MICs of resistant isolates ranging from 1 μg/ml or above. Cross-resistance to polyenes or azoles was not observed and some cases responded positively to subsequent treatment with amphotericin B, or an azole or a combination of both (Pelletier et al., 2005; Prabhu and Orenstein, 2004). The underlying clinical settings in the breakthrough infections range from AIDS to acute myeloid leukaemia and the type of infections reported include oesophagitis, candidaemia and disseminated disease. *Candida* species which have demonstrated resistance to the echinocandins include *C. albicans, C. glabrata, C. parapsilosis, C. krusei, C. guilliermondii* and *C. tropicalis*. In a number of the studies the gene encoding the echinocandin target Fks1 or the alternative Fks2 glucan synthase sub-unit was sequenced and found to contain at least one mutated allele. Risk factors associated with clinical failure include prolonged therapy (Thompson III et al., 2008), in several cases caspofungin had been administered for a long period >15 days (Table 1). The bioavailability of the echinocandins in some niches in the body has also been questioned, for example vitreous penetration (Gauthier et al., 2005) and in the brain (Pelletier et al., 2005). There are also reports of echinocandin resistant moulds with two case reports of patients with haematological malignancies failing to respond to anidulafungin treatment for proven and suspected aspergillosis (Wetzstein et al., 2007). The *FKS* gene sequences of these resistant moulds have not been reported. In another report of a double *C. albicans/A. fumigatus* breakthrough infection the *FKS1* gene of the resistant *A. fumigatus* isolate did not contain any mutations (Arendrup et al., 2009). Another caspofungin resistant *A. fumigatus* isolate was also shown to have a wild-type *FKS1* gene but had altered expression of *FKS1* highlighting another possible resistance mechanism (Arendrup et al., 2008).

## 6 Echinocandin paradoxical growth

*In vitro* studies that test the susceptibility of clinical fungal isolates to echinocandins have reported a phenomenon termed the Eagle effect or the paradoxical growth effect (Wiederhold, 2009). In these studies echinocandins inhibited growth of *C. albicans* at low concentrations but at concentrations from 8–32 μg/ml some growth was observed and at even higher concentrations growth inhibition was achieved again (Stevens et al., 2004, 2006; Wiederhold et al., 2005). This paradoxical growth at supra MICs differs from the trailing growth phenomena observed in some clinical isolates that are capable of persistent but reduced growth at higher drug concentrations (Jacobsen et al., 2007).

The echinocandin paradoxical growth effect varies between *Candida* species. Paradoxical growth occurs most frequently during caspofungin treatment and is most prominent in clinical isolates of *C. parapsilosis*, *C. albicans*, *Candida dubliniensis*, *C. tropicalis* and occasionally with *C. krusei* (Chamilos et al., 2007; Fleischhacker et al., 2008; Stevens et al., 2004). Paradoxical growth was only observed in *C. tropicalis* and *C. krusei* with micafungin and in *C. albicans* and *C. tropicalis* with anidulafungin. Paradoxical growth was not observed when isolates of *C. glabrata* were treated with the echinocandins (Chamilos et al., 2007). This suggests that some *Candida* species are better able to adapt to cell wall damage caused by the echinocandins. Interestingly, paradoxical growth was observed more often with *C. dubliniensis* than with *C. albicans* and more frequently with caspofungin over anidulafungin and micafungin (Fleischhacker et al., 2008; Jacobsen et al., 2007). Using the CLSI M27-A method Fleischhacker et al. (2008) were unable to detect any paradoxical growth in the presence of high concentrations of anidulafungin in either species. Paradoxical growth was also detected by the EUCAST method for some clinical isolates but not by the CLSI M27-A method (Jacobsen et al., 2007). These studies and the inability to reproducibly demonstrate that the paradoxical growth effect occurs *in vivo* have raised the question whether this phenomenon is an artifact of *in vitro* growth (Jacobsen et al., 2007; Wiederhold, 2009). To date there is no clinical evidence that paradoxical growth contributes to resistance or breakthrough infections.

It remains to be determined whether activation of chitin synthesis or paradoxical growth could result in an alternative mechanism of resistance to caspofungin *in vivo*. In one study four clinical isolates of *C. albicans*, which showed the paradoxical effect *in vitro* were subsequently tested *in vivo* to determine whether the same pattern of re-growth was observed (Clemons et al., 2006). By determining CFU from the kidneys of infected mice, caspofungin was found to be efficacious against three of the four clinical isolates (Clemons et al., 2006). The fourth isolate appeared to show paradoxical growth *in vivo*, as it was able to survive better at 20 mg/kg caspofungin than 5 mg/kg caspofungin, although this result could not be reproduced in a subsequent experiment (Clemons et al., 2006).

In contrast there is some evidence to suggest that paradoxical growth occurs *in vivo* when *A. fumigatus* is treated with an echinocandin drug. For example, in a rabbit model of invasive pulmonary aspergillosis, pulmonary infarct scores did not differ between control animals and animals treated with caspofungin at 3 and 6 mg/kg/day, although there was a reduction at 1 mg/kg/day (Petraitiene et al., 2002). Likewise in the same model of infection, lungs from animals treated with up to 2 mg/kg/day of micafungin showed no difference in lung weight, displayed a hyphal fragmentation pattern that was dose-dependent and resulted in increased pulmonary fungal burden compared to the untreated control (Petraitis et al., 2002). In another study that measured fungal burden using quantitative real-time PCR, signs of paradoxical growth were detected in a murine model of invasive pulmonary aspergillosis in response to caspofungin treatment (Wiederhold et al., 2004). Likewise, a murine model of central nervous system aspergillosis demonstrated that treatment with micafungin at 5 mg/kg/day resulted in significant clearance of fungal burden from kidneys, compared to the infected control. In contrast, treatment with micafungin at 10 mg/kg/day did not significantly clear fungal burden from infected kidneys compared to the control (Clemons et al., 2005). Paradoxical growth was also demonstrated in a neutropenic rabbit model of invasive pulmonary aspergillosis treated with anidulafungin. In this case lung weights of animals treated with 10 and 20 mg/kg/day did not differ significantly in weight from lungs of the control (Petraitis et al., 1998). It is worth noting that in the cases mentioned above evidence of paradoxical growth did not result in increased mortality. Although, in one instance treatment with 20 mg/kg/day micafungin, in a murine model of invasive pulmonary aspergillosis, resulted in a higher mortality rate and increased fungal burden. In contrast animals treated with 10 mg/kg/day micafungin showed increased survival (Lewis et al., 2008).

## 7 Biofilms

Treatment of systemic, invasive *Candida* infections is often complicated by the ability of *Candida* spp. to form biofilms that are recalcitrant to azole antifungal drugs. As a fungal biofilm develops and matures extracellular matrix is produced that may act as a physical barrier, protecting the underlying cells and binding antifungal drugs hence lowering the available drug concentration (d’Enfert, 2009). The biofilm extracellular matrix remains poorly characterised but there is evidence that β-(1,3)-glucan is a major component (Al Fattani and Douglas, 2006; Nett et al., 2007). This may be why echinocandins have been shown to be effective against biofilms (Katragkou et al., 2008; Kuhn et al., 2002; Ramage et al., 2001). Furthermore the echinocandins are apparently not substrates for the drug efflux pumps that can be upregulated when *C. albicans* grows as a biofilm (Andes et al., 2004; Mukherjee et al., 2003; Ramage et al., 2002). Upregulation of efflux pumps is a well established mechanism of azole resistance (Sanglard and Odds, 2002). The paradoxical growth observed in planktonic cells has also been seen when cells are grown as biofilms *in vitro* for *C. albicans, C. tropicalis, C. parapsilosis, C. metapsilosis* and *C. orthopsilosis* (Ferreira et al., 2009; Melo et al., 2007).

## 8 Genome-wide screens to identify genes regulated by or involved in the echinocandin response

A number of genome-wide approaches have been applied to study the response of fungal cells to the echinocandin drugs and to identify genes required for adaptive growth in the presence of sub-lethal concentrations of echinocandins. Transcript profiling of cells challenged with sub-MIC concentrations of caspofungin using DNA microarrays have highlighted the genes that are activated in response to echinocandins in *S. cerevisiae, A. niger* and *C. albicans* (Agarwal et al., 2003; Bruno et al., 2006; Liu et al., 2005; Markovich et al., 2004; Meyer et al., 2007; Rauceo et al., 2008). Included in the upregulated genes are those that are a typical signature of activation of the PKC integrity pathway. Indeed this signalling pathway is a major player in the response to echinocandins, indicated by detection of the phosphorylated form of the PKC pathway MAP kinase, Slt2/Mpk1 in *S. cerevisiae,* and Mkc1 in *C. albicans* in response to caspofungin treatment (Reinoso-Martin et al., 2003; Walker et al., 2008). In addition the *mkc1*Δ mutant of *C. albicans* is hypersensitive to caspofungin and does not display the paradoxical growth phenotype *in vitro* (Walker et al., 2008; Wiederhold et al., 2005).

A screen of *C. albicans* transcription factor mutants for altered susceptibility to caspofungin identified a novel protein Cas5 as important for the response to echinocandins and a number of caspofungin responsive genes are regulated by Cas5 (Bruno et al., 2006). In addition, Sko1, a transcription factor downstream of the HOG signalling pathway plays a role in the response to caspofungin but does this in a Hog1 dependent manner (Rauceo et al., 2008). A screen of kinase mutants identified the Psk1 kinase as activating Sko1 in response to caspofungin treatment in a novel signalling pathway (Rauceo et al., 2008).

In *C. albicans* a fitness screen of nearly 3000 heterozygous mutants lacking one copy of each gene, termed a haploinsufficiency screen, was used to investigate the mode of action of novel compounds with potential antifungal activity (Xu et al., 2007). As a proof of concept antifungal drugs with known modes of action, including caspofungin, were also tested. Heterozygous mutants of the β-(1,3)-glucan synthase sub-unit Fks1 and its regulatory sub-unit Rho1 displayed haploinsufficiency, i.e. reduced fitness upon caspofungin treatment.

## 9 Cell wall remodelling and impact of echinocandin-treatment on the immune response

β-(1,3)-glucan is a hallmark component of nearly all fungi. It is not surprising therefore that the human immune system has evolved pattern recognition receptors to β-glucan that signal the presence of a possible invasive fungus and elicit signalling events leading to the induction of the innate immune response. Dectin-1 is a C-type lectin that is expressed in monocytes/macrophages, neutrophils and dendritic cells, which recognises β-(1,3)-glucan of fungal cell walls, plants and some ligands on mycobacterial cells (Netea et al., 2008; Reid et al., 2009; Taylor et al., 2007; van de Veerdonk et al., 2008). Dectin-1 mediated recognition often involves the cooperative binding with other ligands via receptor complexes – most notably TLR2. Binding of β-(1,3)-glucan induces a wide range of responses including the synthesis of cytokines and chemokines and dendritic cell maturation (Reid et al., 2009). As β-(1,3)-glucan is a powerful inducer of the inflammatory innate immune response it has been argued that the role of some of the superficial cell wall layers is to act as a protective barrier to prevent immune cell recognition (Rappleye et al., 2007; Rappleye and Goldman, 2008; Wheeler and Fink, 2006). Because echinocandins have the potential to alter the amount and integrity of both β-(1,3)-glucan and the cell wall, they also have the potential of influencing the immune response to fungi (Ben Ami et al., 2008).

It has been observed that echinocandin-treatment can unmask β-glucan in the deep layers of the cell wall. Unmasking has been demonstrated by showing that impermeable molecules such as the dectin-1-receptor or anti-β-glucan antibody can bind echinocandin-treated cells (Wheeler et al., 2008). Echinocandin-treatment can lead to enhanced killing of fungal cells by phagocytes and alterations in cytokine production by immune cells encountering echinocandin-treated fungi (Chiller et al., 2001; Hohl et al., 2008; Lamaris et al., 2008; Wheeler and Fink, 2006). For example, caspofungin treatment was shown to enhance neutrophil killing of hyphae of *A. fumigatus*, *Rhizopus oryzae*, *Fusarium oxysporum* and *Scedosporium* species (Lamaris et al., 2008). Caspofungin or micafungin treatment of conidia and germlings of *A. fumigatus* led to a reduction in β-(1,3)-glucan levels and reduced synthesis of TNF and CXL2 by macrophages. In contrast, macrophages that were exposed to echinocandin-treated hyphae generated an enhanced cytokine response and had increased β-glucan exposure (Hohl et al., 2008). Hyphae, and not yeast cells, of *C. albicans* are preferentially unmasked by caspofungin treatment *in vivo* (Wheeler et al., 2008).

Therefore echinocandin exposure can cause numerous alterations in the immune response – often enhancing dectin-1 mediated and opsonic recognition of fungal cells, increasing the inflammatory response and inducing more efficient killing by lymphoid cells. The underlying mechanisms are not completely clear. Some of these effects may relate to the general perturbation in cell wall structure and more efficient dectin-1 mediated recognition of β-glucan. Echinocandins may also reduce β-glucan content of the cell wall even when dectin-1 mediated recognition is apparently enhanced.

## 10 Induction of chitin biosynthesis by cell wall salvage pathways and the potential of combination therapies

**Fig. 3:**
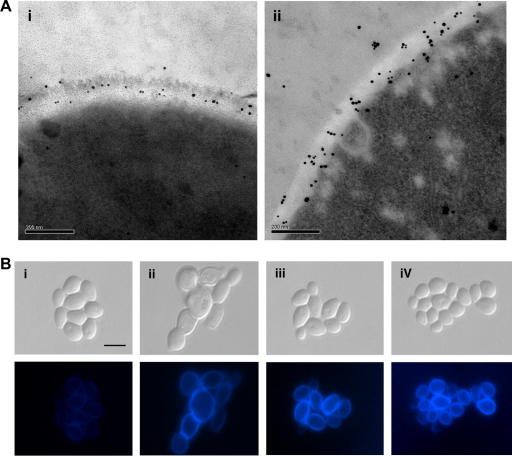
Treatment with caspofungin increases chitin content in *C. albicans*. (A) Transmission electron micrographs showing WGA-colloidal gold staining of chitin in wild-type *C. albicans* (i) and after treatment with a sub-MIC concentration (0.032 μg/ml) of caspofungin (ii). (B) CFW staining was used to assess chitin levels of cells grown in YPD alone (i), after treatment with 0.032 μg/ml caspofungin (ii), in YPD with 200 mM CaCl_2_ and 100 μg/ml CFW (iii) and after pre-growth with CaCl_2_ and CFW followed by exposure to 0.032 μg/ml caspofungin (iv). DIC images (top panels) and CFW fluorescent images (bottom panels). Scale bars are (A) 0.2 μm and (B) 2 μm.

The fungal cell wall is a dynamic structure and preventing the synthesis of one component of the cell wall is known to lead to a compensatory increase in another. Consequently, inhibition of β-(1,3)-glucan synthesis by caspofungin results in a compensatory increase in chitin synthesis in *C. albicans* (Fig. 3) (Walker et al., 2008). This activation of chitin synthesis is mediated by the PKC, Ca^2+^–calcineurin and HOG signalling pathways (Munro et al., 2007). Furthermore, treating cells with activators of the PKC and Ca^2+^–calcineurin pathways (Calcofluor White and Ca^2+^) elevates chitin content and reduces susceptibility to caspofungin in *C. albicans* and *A. fumigatus* (Walker et al., 2008; Walker, Munro, Gow, unpublished). Similarly, *C. albicans* cell wall mutants with elevated chitin contents are less susceptible to caspofungin (Plaine et al., 2008). At high concentrations of caspofungin *C. albicans* can form resistant colonies, which have a significantly higher chitin content than susceptible, wild-type cells. This may constitute a form of drug tolerance and appears to be an adaptive response as sub-culturing these resistant cells in the absence of caspofungin resulted in the chitin content returning to wild-type levels (Walker et al., 2008). This suggests that *C. albicans* has the natural ability to adapt to caspofungin treatment through a compensatory elevation of chitin content.

The elevated chitin response is not specific to *C. albicans* as clinical isolates of *C. tropicalis*, *C. parapsilosis*, *C. guilliermondii* and *A. fumigatus* also show an increase in chitin content in response to treatment with caspofungin (Fortwendel et al., 2009; Walker, Gow, Munro, unpublished). Furthermore these species display the highest incidence of *in vitro* paradoxical growth, in response to caspofungin treatment (Antachopoulos et al., 2008; Chamilos et al., 2007). In contrast, isolates of *C. glabrata* and *C. krusei* demonstrate little or no occurrence of paradoxical growth and consequently no increase in chitin content was observed after treatment with caspofungin in these species (Chamilos et al., 2007; Walker, Gow, Munro, unpublished). However, increased *SLT2* expression and chitin content have been associated with incomplete killing of *C. glabrata* by caspofungin (Cota et al., 2008). Therefore, elevation of chitin content in response to caspofungin treatment is a good indication of which species are prone to paradoxical growth. Interestingly isolates of different *Candida* species that have point mutations in the Fks1 hot spot regions, rendering them resistant to caspofungin, do not show a compensatory increase in chitin content in response to caspofungin treatment (Walker, Gow, Munro, unpublished). Instead, isolates with point mutations in Fks1 often contain a higher basal chitin content compared to isolates with wild-type Fks1 (Walker, Gow, Munro, unpublished).

*Candida* species and *A. fumigatus* demonstrate a compensatory increase in chitin content in response to caspofungin treatment highlighting the potential of combining chitin synthase inhibitors with the echinocandins for improved and/or broader spectrum therapy. Likewise, the response to caspofungin in *C. albicans* and *A. fumigatus*, and the paradoxical growth that may be observed, have been shown to involve the PKC, Ca^2+^–calcineurin and HOG signalling pathways (Cota et al., 2008; Fortwendel et al., 2009; Kelly et al., 2009; Walker et al., 2008; Wiederhold et al., 2005). Consequently, inhibitors of these pathways may also act synergistically with caspofungin. Recently the Cap1 signalling pathway has also been shown to be activated in response to caspofungin treatment in *C. albicans* (Kelly et al., 2009).

Chitin synthase inhibitors have been shown to enhance the activity of caspofungin against *C. albicans* and *A. fumigatus* (Sandovsky-Losica et al., 2008; Steinbach et al., 2007a; Walker et al., 2008). This is an echinocandin wide effect as nikkomycin Z also enhances the activity of micafungin against *A. fumigatus* (Ganesan et al., 2004). Similarly, anidulafungin shows synergy with nikkomycin Z against clinical isolates of *A. fumigatus*, *C. albicans*, *Rhizopus* spp. and *Coccidioides immitis* (Stevens, 2000). Combination treatment with chitin inhibitors and the echinocandins would be particularly beneficial in the treatment of isolates which have intrinsic resistance to the echinocandins. For example, the echinocandins typically display fungistatic activity against *A. fumigatus*. In contrast, combination treatment of *A. fumigatus* with nikkomycin Z and the echinocandins leads to synergistic killing through formation of extremely swollen spores with aberrant walls which are prone to lysing (Chiou et al., 2001; Fortwendel et al., 2009; Steinbach et al., 2007a; Walker, Munro, Gow, unpublished). Furthermore, growth of a *C. albicans* strain, which was resistant to caspofungin due to homozygous point mutations in *Ca*Fks1, could be inhibited by combined treatment with caspofungin and several different chitin inhibitors (Walker et al., 2008). Reflecting the *in vitro* results, treatment with micafungin significantly prolonged survival when used in combination with nikkomycin Z against pulmonary and systemic murine aspergillosis (Clemons and Stevens, 2006; Luque et al., 2003). Currently there are no chitin inhibitors, which are approved for clinical use, although there is obvious potential for combination therapy with the echinocandins and chitin inhibitors.

A blockade of the calcineurin pathway, through disruption of genes or by use of calcineurin inhibitors, has also been shown to act synergistically with caspofungin against *C. albicans*, *A. fumigatus* and *C. neoformans*, *in vitro* (Del Poeta et al., 2000; Fortwendel et al., 2009; Kontoyiannis et al., 2003; Kraus and Heitman, 2003; Steinbach et al., 2007a; Walker et al., 2008). Combined treatment of *C. albicans* with caspofungin and the calcineurin inhibitor, cyclosporine, also prevented the paradoxical growth seen at high concentrations of caspofungin (Wiederhold et al., 2005).

Other potential mechanisms have been implicated in resistance to the different classes of antifungal drugs but have been best studied with the azoles (Sanglard and Odds, 2002). Azole resistance can be conferred by altering the drug target, Erg11, by changing Erg11 expression levels through a variety of mechanisms or upregulation of efflux pumps, Mdr1, Cdr1 and Cdr2. In comparison resistance to echinocandins is acquired by altering its target but there is little evidence so far to suggest that the other mechanisms are occurring. The role of Cdr1 and Cdr2 in echinocandin susceptibility remains under debate (Niimi et al., 2006; Schuetzer-Muehlbauer et al., 2003). Altered expression of the echinocandin target in response to drug has not been studied in detail. However, *C. glabrata* clinical isolates with hot spot mutations in Fks1 had elevated *FKS1* expression (Garcia-Effron et al., 2009a) suggesting this mechanism may also be occurring in at least some resistant isolates.

## 11 Conclusion

Echinocandins are an important new generation of antifungal agent whose mode of action is to bind to Fks1 leading to reduced β-(1,3)-glucan synthesis resulting in damage to the integrity of the cell wall. Clinical resistance to this class of agent is rare, although point mutations in Fks1 in a range of species of fungi have been found to confer resistance to echinocandins that can result in clinical failures. In addition exposure to echinocandins induces a salvage mechanism involving the upregulation of chitin synthesis. This physiological adaptation enables a fungus to survive at otherwise lethal concentrations of echinocandin and can be described as a tolerance mechanism. Recently discovered mechanisms of resistance to echinocandins also point the way to opportunities to enhance the efficacy of this class of drug via combination therapies that prevent these salvage mechanisms coming into operation. In addition, perturbation of β-glucan synthesis in fungi leads to enhanced killing by phagocytes of the lymphoid cells of the immune system and alterations in cytokine production – probably by unmasking β-(1,3)-glucan that is normally buried in the internal layers of the cell wall.

## Figures and Tables

**Table 1:** Selected cases of echinocandin therapy failure.

Clinical setting	Pathogen	Echinocandin therapy & duration	CAS MIC (μg/ml)	*FKS1* mutation	Reference
Oesophagitis (AIDS)	*C. albicans*	CAS (2 courses)	>64	ND	Hernandez et al. (2004)
Prosthetic aortic valve endocarditis	*C. parapsilosis*	CAS + FCZ for 6 weeks	>16	ND	Moudgal et al. (2005)
Disseminated (abdominal surgery)	*C. krusei*	CAS for 15 days	2	ND	Pelletier et al. (2005)
Disseminated	*C. albicans*	CAS	4	S645F	Park et al. (2005)
Candidaemia	*C. krusei*	CAS	32	R1361G	Park et al. (2005)
Candidaemia	*C. glabrata*	CAS, AMB, VRC,	>8	S633P	Dodgson et al. (2005)
Azole refractory oesophagitis (AIDS)	*C. albicans*	CAS then MFG for total of 10 months	2	S645F, R1361H	Laverdiere et al. (2006)
Recurrent oesophagitis (AIDS)	*C. albicans*	CAS, 2 courses with dose escalation	8	S645P (homo)	Miller et al. (2006)
Candidaemia (Acute Myeloid Leukaemia)	*C. krusei*	CAS for 17 days	8	F655C (Het)	Hakki et al. (2006) and Kahn et al. (2007)
Candidaemia	*C. glabrata*	CAS (3 courses)	>16	ND	Daneman et al. (2006)
Candidaemia	*C. parapsilosis*	CAS (+phenytoin)	0.25	ND	Cheung et al. (2006)
Not reported	*C. glabrata*	Not reported	4	F659 V of Fks2	Katiyar et al. (2006)
Candidaemia	*C. glabrata*	VRC, CAS for 136 days	>8	ND	Krogh-Madsen et al. (2006)
Oesophagitis (AIDS)	*C. albicans*	CAS, 2 courses	2	F641S	Baixench et al. (2007)
Oesophagitis (AML)	*C. tropicalis*	CAS	4	ND	Pasquale et al. (2008)
Candidaemia	*C. glabrata*	CAS	>4	D632E	Cleary et al. (2008)
Candidaemia	*C. glabrata*	CAS for 61 days	8	F659 V of Fks2	Thompson III et al. (2008)
Candidaemia (HSCT)	*C. parapsilosis*	CAS for 41 days	1	ND	Kabbara et al. (2008)
Candidaemia (HSCT)	*C. parapsilosis*	CAS for 50 days	1	ND	Kabbara et al. (2008)
Candidaemia (HSCT)	*C. guilliermondii*	CAS for 26 days	0.5	ND	Kabbara et al. (2008)
Candidaemia (AML)	*C. tropicalis*	CAS for 16 days	4	S645P (Het)	Garcia-Effron et al. (2008b)
Candidaemia (HSCT)	*C. tropicalis*	CAS for 44 days	4	S645P (Het)	Garcia-Effron et al. (2008b)
Candidaemia (cancer)	*C. tropicalis*	CAS for 21 days	1	F641L (homo)	Garcia-Effron et al. (2008b)
Disseminated	*C. albicans*	CAS for 34 days	1 (>32 Etest)	S645P	Arendrup et al. (2009)
Not reported	*C. glabrata*^a^	CAS therapy or prophylaxis	>2	Fks1,2^a^	Garcia-Effron et al. (2009a)

a Twelve different clinical isolates reported with various Fks1, Fks2 point mutations.
